# Multi-omic analysis of chronic myelomonocytic leukemia monocytes reveals metabolic and immune dysregulation leading to altered macrophage polarization

**DOI:** 10.1038/s41375-024-02511-4

**Published:** 2025-01-15

**Authors:** Hasse M. Addinsell, Rachel Cant, Nathan J. Hull, Yu-Hung Wang, Tim C. P. Somervaille, Daniel H. Wiseman, Kiran Batta

**Affiliations:** 1 Epigenetics of Haematopoiesis Group, Division of Cancer Sciences, The University of Manchester, Manchester, UK; 2 Leukaemia Biology Laboratory, Cancer Research UK Manchester Institute, The University of Manchester, Manchester, UK; 3 The Christie Hospital NHS Foundation Trust, Manchester, UK

**Keywords:** Leukaemia, Myelodysplastic syndrome

## To the Editor:

Chronic myelomonocytic leukemia (CMML) is a clonal myelodysplastic/myeloproliferative neoplasm characterized by persistent peripheral blood (PB) monocytosis with few effective treatment options [1]. CMML is genetically homogeneous, with the most commonly mutated gene class encoding epigenetic regulators, suggesting epigenetic dysregulation as a core hallmark of CMML biology [2]. However, few CMML-specific epigenomic studies have been performed, and all focused on whole bone marrow mononuclear cells (BM-MNCs). Since transcriptional networks and enhancer landscapes are cell type-specific, analyzing sorted leukemic cell populations could reveal tractable vulnerabilities.

Although a stem cell disease, monocytosis is CMML’s defining clinicopathological feature, with expanded clonal monocytes driving many aspects of morbidity [3]. Importantly, monocytes form part of the microenvironment, wherein they contribute to leukemia progression [4]. Thus, strategies to target malignant monocytes could hold wide therapeutic value. An understanding of the epigenetic regulatory networks mediating the survival and differentiation of CMML monocytes could reveal novel therapeutic vulnerabilities. Thus, we profiled the epigenetic and transcriptional signatures specific to the monocyte compartment in CMML.

**Fig. 1:**
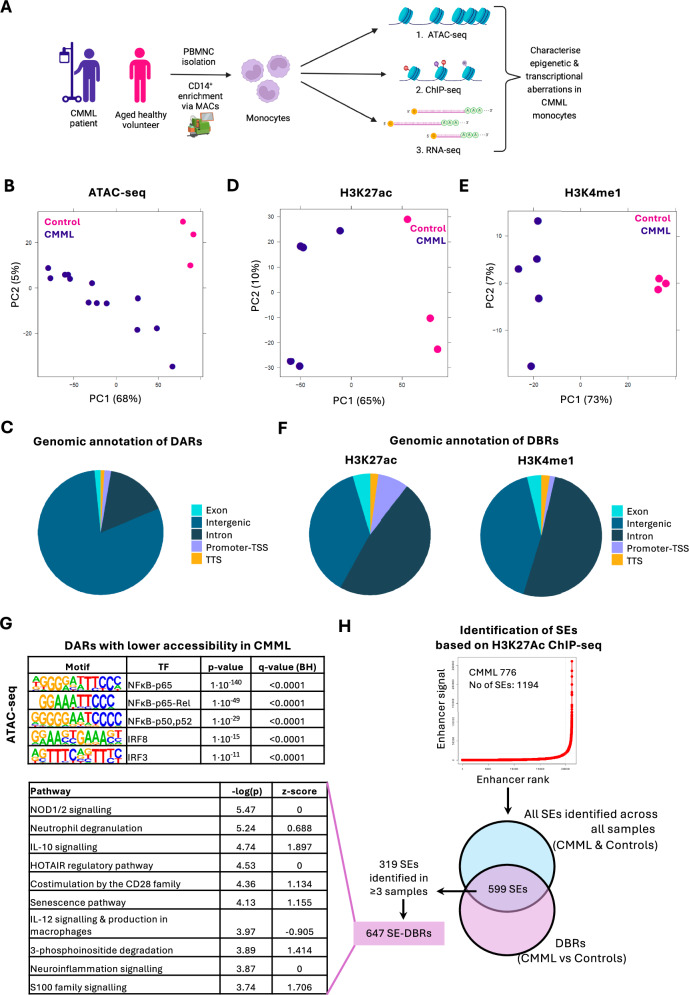
CMML monocytes display enhancer dysregulation. **A** Schematic outline of the study investigating altered epigenetic and transcriptional networks involved in CMML monocytes. **B** Principal component analysis (PCA) of ATAC-Seq data. **C** Genomic annotation of differentially accessible regions (DARs). **D**, **E** PCA of H3K27ac (**D**) and H3K4me1 (**E**) ChIP-seq data. **F** Genomic annotation of H3K27ac (left) and H3K4me1 (right) differentially bound regions (DBRs). **G** Motifs were most significantly enriched in DARs with lower accessibility in CMML compared to healthy controls. TF transcription factor, BH Benjamini–Hochberg correction. **H** Diagram illustrating Super Enhancer (SE) analysis workflow. SEs were called with the ROSE algorithm (CMML sample 776 shown as an example) and filtered for overlap with H3K27ac DBRs (599 SEs). Then, we focused on SEs overlapping DBRs found in ≥3 samples (319 SEs). The H3K27ac DBRs (647, SE-DBRs) that were part of 319 SEs were then mapped to genes. Pathway analysis performed (shown on the left) on genes that SE-DBRs mapped to, performed with ingenuity pathway analysis (IPA).

CD14+ monocytes isolated from CMML patients and control age-matched healthy volunteers were subjected to ATAC-seq, ChIP-seq for histone-marks (H3K27ac/H3K4me1/H3K4me3/H3K27me3) and RNA-Seq (Fig. 1A and Supplementary Table 1). Principal component analysis (PCA) of ATAC-Seq data revealed substantial epigenetic heterogeneity amongst CMML samples, with most differentially accessible regions (DARs) mapping to intergenic/distal elements, suggesting enhancer dysregulation (Fig. 1B, C and Supplementary Table 2). Most significant DARs lost accessibility in CMML, mapping to genes involved in monocyte activation and inflammatory responses. Despite the significant correlation between fold changes in DARs and differentially expressed genes (DEGs), only 85 DARs were linked to DEGs, suggesting that additional factors may be required to alter gene expression or the poised state of distal elements (Supplementary Fig. 1A). To further investigate dysregulated epigenetic networks, we analyzed histone modification patterns characteristic of bivalency (H3K27me3/H3K4me3) and putative transcriptional enhancers (H3K4me1/H3K27ac). Global analysis of each revealed significant differences supporting widespread epigenetic dysregulation (Fig. 1D, E and Supplementary Fig. 1B, C). Unlike H3K27me3 and H3K4me3 profiles, we observed profound alterations in H3K27ac and H3K4me1 marks in CMML monocytes. Genomic annotation of the H3K27ac and H3K4me1 differentially bound regions (DBRs) revealed these as mostly intergenic or intronic (Fig. 1F and Supplementary Tables 3,4), again consistent with enhancer dysregulation. Generally, we observed good correlation between changes in H3K27Ac occupancy and chromatin accessibility (Supplementary Fig. 1D). Together these data suggest that CMML monocytes display genome-wide alterations in chromatin modifications and accessibility, predominantly at sites of putative transcriptional enhancers.

We next performed motif analysis on DBRs and DARs to elucidate TF networks affected by epigenetic dysregulation. This revealed strong enrichment for binding motifs of the canonical p65 subunit of the inflammatory regulator NF-κB in regions with decreased accessibility in CMML (Fig. 1G); surprising given the purported proinflammatory microenvironment in CMML [5]. Conversely, enrichment for ZAC1, STAT6 and ZFX motifs was observed in DARs with increased chromatin accessibility in CMML monocytes (Supplementary Fig. 2A). Notably, ZAC1 has been shown to suppress NF-κB activity by inhibiting p65-phosphorylation [6]. To identify putative oncogenic drivers, we defined super-enhancers (SEs) based on H3K27ac ChIP-seq data using the ROSE algorithm, identifying a mean of 874 SEs per sample (Fig. 1H). Focusing on SEs that overlapped DBRs revealed 599 SEs as differentially bound (FDR < 0.05). Of these, 319 were shared between ≥3 CMML samples, mapping to 647 DBRs (SE-DBRs). Pathway analysis of genes mapping to SE-DBRs revealed enrichment for several pathways dysregulated in CMML, including IL-10^5^ and S100 family signaling (Fig. 1H). In line with epigenetic suppression of NF-κB binding sites in CMML monocytes, we identified a SE near the gene encoding the NF-κB regulator *NFKBIZ*, whose expression was also downregulated in CMML (Supplementary Fig. 2B, C). Thus, a subset of NF-κB binding sites are epigenetically repressed in CMML monocytes.

**Fig. 2:**
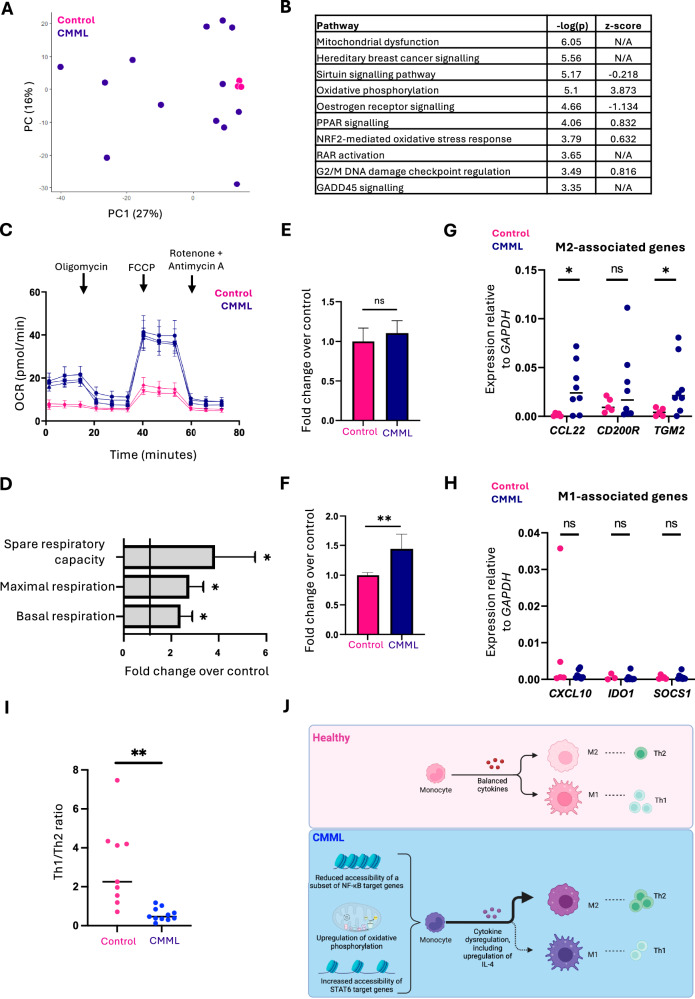
Metabolically dysregulated CMML monocytes show a bias towards an M2 phenotype, and this is accompanied by a shift towards Th2 cells. **A** PCA of RNA-seq data. **B** Pathway analysis of RNA-seq data, performed with IPA. Top ten pathways (based on *p* value) are shown. **C** Oxygen consumption rate (OCR) data for mitochondrial stress test. Representative graph shown (see also Supplementary Fig. 4). Data were normalized using DNA content as determined by CyQUANT assay and multiplied by a scale factor. **D** Change in selected respiratory parameters in CMML monocytes relative to healthy monocytes, as determined by mitochondrial stress tests (*N* = 7, CMML; *N* = 4, Controls). *p < 0.05. **E**, **F** MitoTracker Green FM (**E**) and MitoTracker Red CMXRos (**F**) fluorescence in CMML and control monocytes, as determined by flow cytometry. Data were plotted as fold change in mean fluorescence intensity over average signal in healthy controls (*N* = 7, CMML; *N* = 4, Controls). **p < 0.01. **G**, **H** Quantitation of expression levels of genes associated with the M2 (**G**) or M1 (**H**) phenotype in day 5 M0 monocyte-derived macrophages (MDMs) by qRT-PCR. **I** Th1/Th2 ratio in control and CMML PBMNCs at day 0, as determined by flow cytometry. **p < 0.01. **J** Working model: Healthy monocytes can differentiate towards either M2 (“anti-inflammatory”) or M1 (“proinflammatory”) macrophages, which is regulated by cytokine milieu. CMML monocytes exhibit an innate bias towards an M2 macrophage phenotype, which is presumably mediated by the cytokine imbalance observed in CMML plasma and cell-intrinsic features. This bias towards M2 macrophages then translates into a relative increase in Th2 cells at the expense of Th1 cells.

RNA-seq identified 879 DEGs in CMML: 430 upregulated and 449 downregulated (Supplementary Table 5). The top upregulated genes (e.g., *SLC2A5*, *EGFL7*) are known putative therapeutic targets in AML. PCA revealed marked transcriptional heterogeneity amongst CMML, mirroring the epigenetic heterogeneity (Fig. 2A). Ingenuity Pathway Analysis revealed significant enrichment in CMML for “mitochondrial dysfunction” and “oxidative phosphorylation (OxPhos)”, amongst others (Fig. 2B). Contrasting with a previous study [7] we did not observe an enrichment of inflammatory pathways. We thus specifically investigated the expression of five genes from the proinflammatory signature defined in that study. Only one patient showed high expression (Supplementary Fig. 3A), suggesting heterogeneity of inflammatory signatures. Gene set enrichment analysis identified pathways related to mitochondrial energy metabolism as variously disrupted in CMML monocytes (Supplementary Fig. 3B). Re-analysis of published transcriptome data from CMML monocytes [8] revealed similar enrichment for “fatty acid metabolism” and “oxidative phosphorylation” (Supplementary Fig. 3C).

To functionally validate increased OxPhos activity, we performed mitochondrial stress tests on CMML and control monocytes (Fig. 2C and Supplementary Fig. 4). Basal respiration, maximal respiration, and spare respiratory capacity were all significantly increased in CMML (Fig. 2D), contrasting with low baseline levels of OxPhos in controls. To investigate whether increased OxPhos capacity was a result of increased mitochondrial mass, flow cytometry was performed, using: MitoTracker Green FM, whose uptake represents a proxy for mitochondrial mass; and MitoTracker Red CMXRos, uptake of which is linked to mitochondrial membrane potential ΔΨ_mt_. CMML monocytes did not exhibit changes in total mitochondrial mass (Fig. 2E). However, they exhibited hyperpolarization of ΔΨ_mt_ (Fig. 2F); likely resulting from the increased OxPhos in CMML monocytes, since activity of the electron transport chain releases protons into the intermembrane space. Thus, transcriptional dysregulation of OxPhos genes translates into increased respiratory capacity in CMML monocytes, highlighting a potential therapeutically targetable dependency. Drugs targeting mitochondrial metabolism have attracted considerable interest but have been hampered by inadequate potency and off-target effects, necessitating the development of more selective OxPhos inhibitors.

We next investigated the functional significance of reduced chromatin accessibility at NF-κB binding sites in CMML monocytes. The lack of corresponding changes in inflammatory gene expression suggests an epigenetic phenotype that might affect cells’ potential to respond to inflammatory stimuli or differentiation cues. The observed profile is reminiscent of the signature seen in tolerant monocyte-derived macrophages (MDM), which are unresponsive to proinflammatory stimuli and show bias towards M2 phenotye [9]. Pathway analysis of genes with putative enhancers containing NF-κB binding sites with lower accessibility in CMML showed strong enrichment for the macrophage classical activation signaling pathway (M1), suggesting repression of this pathway (Supplementary Fig. 5A). In contrast, genomic regions that gain chromatin accessibility showed strong enrichment for STAT6 binding motifs, a TF important in establishing the M2 phenotype [10] (Supplementary Fig. 2A). These results suggest that a subset of proinflammatory NF-κB target genes associated with M1 macrophage priming are epigenetically repressed in CMML, while regions linked to alternative activation of macrophages are accessible, promoting an M2 bias.

To investigate further, primary CMML monocytes were differentiated ex vivo into unpolarized (M0) MDMs (Supplementary Fig. 5B), which expressed significantly higher *CCL22* and *TGM2*, both associated with M2 phenotype, compared with control MDMs (Fig. 2G). Increased expression of the M2-associated gene *CD200R* was also noted, although it did not reach statistical significance. Conversely, there was no difference in the expression of M1-associated genes *CXCL10*, *IDO1*, and *SOCS1* (Fig. 2H). Thus, in the absence of polarizing stimuli, CMML MDMs are transcriptionally biased towards the M2 phenotype; consistent with our observed upregulation of OxPhos and fatty acid oxidation pathways in CMML monocytes (themselves linked to M2 phenotype). In support, unpolarized CMML MDMs displayed higher plastic adherence, elongated morphology, and more/larger vacuolation compared with controls (Supplementary Fig. 5C), all features associated with M2 phenotype [11].

Macrophage polarization is intricately linked to immune responses, including modulating T-helper subset responses. M1 macrophages polarize T-cells towards Th1 or Th17 phenotypes, whilst M2 polarize towards Th2 [12]. We thus measured the Th1/Th2 ratio in PBMNCs, observing a significant decrease in CMML, in keeping with the M2 bias observed in monocytes/MDMs (Fig. 2I). Thus, taken together CMML monocytes are epigenetically, metabolically, and functionally primed towards an M2 macrophage fate, accompanied by an immunological bias towards Th2 polarization (Fig. 2J).

Here we profiled the chromatin accessibility and histone mark epigenome in CMML monocytes and age-matched controls, identifying widespread enhancer dysregulation. Our analyses suggest substantial heterogeneity in the purported inflammatory phenotype, which may not be a consistent feature of CMML monocytes. Our integrated analysis revealed widespread epigenomic dysregulation affecting SEs, notably proinflammatory pathways, in keeping with an observed anti-inflammatory M2 bias in CMML MDMs. In line with previous reports [13], we found a decreased Th1/Th2 ratio in CMML PB. Th1 stimulates the killing activity of macrophages and secrete interferons and TNF, while Th2 secrete IL-4, IL-5, and IL-13 and induce M2 phenotype in macrophages [14]. Thus, a bias towards Th2 may have pleiotropic immunological downstream effects, potentially reinforcing the macrophage polarization bias.

The bias towards M2 macrophages and Th2 cells in CMML suggests a shift towards type 2 immune responses. These are normally associated with tissue repair, but when excessive can have pathological consequences, including tissue fibrosis and autoimmune phenomena [15]. Whether and how this might be pertinent to CMML features, for example BM fibrosis (30% of CMML cases) requires further study. In conclusion, we demonstrate substantial epigenomic dysregulation in CMML monocytes, rewiring regulatory regions including SEs and resulting in epigenetic suppression of a subset of NF-κB target genes. CMML monocytes are epigenetically and metabolically primed towards an M2 macrophage state, with potential downstream immunological and clinical consequences.

## Supplementary information

Supplementary Data

Supplementary Table 2

Supplementary Table 3

Supplementary Table 4

Supplementary Table 5

## Data Availability

Sequencing data were deposited into the Gene Expression Omnibus database under accession numbers GSE283200, GSE283202, and GSE283203.
