# Supplementary material for: Multi-omic analysis of chronic myelomonocytic leukemia monocytes reveals metabolic and immune dysregulation leading to altered macrophage polarization
Source: Leukemia. 2025 Jan 15;39(3):770–4. doi: 10.1038/s41375-024-02511-4 (PMC11879862; doi:10.1038/s41375-024-02511-4)
Supplement: Supplementary file 1 — Supplementary Data [file 41375_2024_2511_MOESM1_ESM.pdf]

Multi-omic analysis of chronic myelomonocytic leukemia monocytes reveals metabolic and immune dysregulation leading to altered macrophage polarization

Hasse M Addinsell[1], Rachel Cant[1], Nathan J Hull[1], Yu-Hung Wang[1], Tim C. P. Somervaille[2,3], Daniel H Wiseman[1,3*], Kiran Batta[1*]

Supplementary Tables:

Supplementary Table 1: Cohort information

ID	Disease state	MD/MP	Age	Sex	Mutational profile	Included in
HB2	Healthy	NA	57	F	ND	ATAC-seq, RNA-seq, ChIP-seq
HB3	Healthy	NA	59	F	ND	ATAC-seq, RNA-seq, ChIP-seq
HB4	Healthy	NA	64	F	ND	ATAC-seq, RNA-seq, ChIP-seq
HB11	Healthy	NA	64	F	ND	Mitochondrial assays
HB12	Healthy	NA	63	F	ND	Mitochondrial assays
HB13	Healthy	NA	52	M	ND	Mitochondrial assays
HB14	Healthy	NA	55	F	ND	Mitochondrial assays
HB19	Healthy	NA	56	F	ND	Macrophage profiling, T cell flow
HB20	Healthy	NA	56	M	ND	T cell flow
HB21	Healthy	NA	58	M	ND	T cell flow
HB22	Healthy	NA	60	M	ND	T cell flow
HB23	Healthy	NA	53	F	ND	T cell flow
HB28	Healthy	NA	53	M	ND	Macrophage profiling, T cell flow
HB29	Healthy	NA	58	F	ND	Macrophage profiling, T cell flow
HB30	Healthy	NA	33	M	ND	Macrophage profiling, T cell flow

HB31	Healthy	NA	58	M	ND	Macrophage profiling, T cell flow
201	CMML-2	MP	73	F	*SRSF2*, *TET2*, *ASXL1*, *NRAS*, *JAK2*, *ETV6*	ATAC-seq, RNA-seq
572	CMML-2	MP	60	M	*BCOR*, *KRAS*, *RUNX1*, *ZRSR2*, *ASXL1*, *TET2*	ATAC-seq, RNA-seq
561	CMML-1	MD	66	M	*TET2*, *ZRSR2*	T cell flow
665	CMML-1	MP	78	M	*SRSF2*, *RUNX1*, *TET2*	ATAC-seq, RNA-seq
666	CMML-1	MD	64	F	*SRSF2*, *TET2*, *ASXL1*, *CBL*	ATAC-seq, RNA-seq
763	CMML-2	MP	69	M	*ASXL1*, *SF3B1*, *NF1*	ATAC-seq, RNA-seq
776	CMML-2	MP	73	M	*SRSF2*, *ASXL1*, *SETBP1*, *CBL*	ATAC-seq, RNA-seq, ChIP-seq
786	CMML-1	MP	74	M	*TET2*, *CBL*, *SRSF2*	ATAC-seq, RNA-seq, ChIP-seq
796	CMML-1	MP	63	F	*TET2*, *KRAS*	ATAC-seq, RNA-seq
812	CMML-2	MP	71	F	*TET2*, *SF3B1*, *RUNX1*	ATAC-seq, RNA-seq, ChIP-seq
822	CMML-2	MP	63	M	*NPM1*, *NRAS*	ATAC-seq, RNA-seq
830	CMML-1	MD	64/65	M	*TET2*, *NRAS*, *CBL*	ATAC-seq, RNA-seq, ChIP-seq, mitochondrial assays
872	CMML-2	MP	71	F	*TET2*, *EZH2*, *RUNX1*, *FLT3*-ITD	ATAC-seq, RNA-seq

932	CMML-1	MP	69	F	*TET2*, *KRAS*	ATAC-seq, RNA-seq, ChIP-seq
1107	CMML-1	MP	79	F	*JAK2*, *TET2*, *ASXL1*, *CBL*	Mitochondrial assays
1115	CMML-1	MP	79	M	*SRSF2*, *TET2*, *RUNX1*, *NRAS*	Mitochondrial assays
1122	CMML-1	MP	64	M	*SRSF2*, *TET2*, *ASXL1*, *RUNX1*, *NRAS*	Macrophage profiling, T cell flow
1188	CMML-1	MP	82	F	*SRSF2*, *TET2*, *KRAS*	Macrophage profiling, T cell flow, mitochondrial assays
1213	CMML-1	MD	56	M	*SRSF2, TET2, NRAS*	Macrophage profiling, T cell flow
1223	CMML-1	MP	74	F	*SRSF2*, *TET2*, *EZH2*	Mitochondrial assays
1236	CMML-1	MP	66	M	*ASXL1*, *EZH2*, *FLT3*	T cell flow
1238	SM-AHN (CMML)	ND	75	M	*SRSF2*, *TET2*, *ASXL1*, *RUNX1*, *NRAS*, *KIT*	Mitochondrial assays
1239	CMML-1	MP	71	F	*TET2*, *ASXL1*, *NRAS*	Mitochondrial assays
1244	CMML-1	MD	60	M	*SRSF2*, *TET2*, *CBL*	Macrophage profiling, T cell flow
1255	CMML-1	MP	76	F	*TET2, CBL*	Macrophage profiling, T cell flow

1266	CMML-1	MP	72	M	*SRSF2, TET2, CBL*	Macrophage profiling, T cell flow
1269	CMML-1	MP	84	M	*NF1, IDH2, ASXL1, SRSF2*	Macrophage profiling, T cell flow
1271	CMML-2	MP	80	F	*TET2, ASXL1, NRAS*	Macrophage profiling, T cell flow
1284	CMML-1	MP	75	M	*U2AF1, FLT3, NRAS, TP53*	T cell flow

ND = not determined; NA = not applicable; SM-AHN = systemic mastocytosis with associated haematological neoplasm. Variants of unknown significance are included in this table.

Supplementary Table 2: List of ATAC-Seq data differentially accessible regions comparing CMML monocytes with controls.

Supplementary Table 3: List of H3K27ac differentially bound regions comparing CMML monocytes with controls.

Supplementary Table 4: List of H3K4me1 differentially bound regions comparing CMML monocytes with controls.

Supplementary Table 5: List of differentially expressed genes comparing CMML monocytes with controls.

Supplementary Methods

Patients and samples: CMML samples were obtained from patients treated at The Christie NHS Foundation Trust (Manchester, UK) recruited to the Manchester Cancer Research Centre Tissue Biobank. Peripheral blood mononuclear cells (PBMNCs) from age matched Healthy volunteers (HVs) were used as controls. All patients and controls gave informed consent according to the Declaration of Helsinki. The study was approved by the UK Health Research Authority (REC: 19/LO/0564). Clinical metadata for the sample cohort are summarized in Supplementary Table 1. For experiments using fresh blood samples, CMML blood was obtained from the Christie hospital (Manchester, UK) and control samples were obtained on the morning of the experiment in Vacutainer blood collection tubes, coated with EDTA (BD).

Primary cell processing and culture: PBMNCs were isolated from fresh whole blood by density gradient separation using Lymphoprep (StemCell Technologies, 07861). In experiments where cryopreserved samples were used, dead cell removal using MACS (Miltenyi Biotec, 130-090-101) was carried out prior to enrichment of the desired population. Enrichment of $CD14^+$ cells (130-050-201) and dead cell removal were carried out using an autoMACS pro (Miltenyi Biotec), according to the manufacturer's instructions. Purity of cell populations enriched for $CD14^+$ cells with MACS was assessed using an Attune NxT acoustic focusing cytometer (Thermo Fisher Scientific), LSR II flow cytometer (BD) or Fortessa X20 flow cytometer (BD). Cells were stained with CD14-FITC antibody (eBioscience 11-0149-42) or CD14-pacific blue antibody (BD 558121). All flow cytometry analysis was carried out using FlowJo (v10.10).

To assess the ratio of Th1 to Th2 cells, flow cytometry was carried out using a Novocyte Quanteon (Agilent) using the following antibodies: CD3-AF700 (300323), CD4-FITC (300505), CD8a-BV510 (301047), CXCR3-BV421 (353715), CCR4-PE-Cy7 (359409), CCR6-PE

(353409), CCR10-APC (341505) (all from Biolegend), fixable near-IR live/dead stain (Thermo Fisher, L34975), and analysed using a previously reported gating strategy[1].

Macrophage differentiation and polarization: Monocytes were cultured in RPMI-1640, supplemented with 10% heat-inactivated FBS, 50 U/mL penicillin and 50 µg/mL streptomycin and 25 ng/mL M-CSF (monocyte medium). To differentiate monocytes into macrophage, cells were maintained in monocyte medium for 5 days. 1 million CD14$^+$ monocytes isolated from fresh blood were seeded per well of a 6-well plate, which was scaled down if cell numbers were limited. Medium was changed every 2-3 days. Macrophages were detached by incubating in PBS with 5 mM EDTA, on ice, for 40 minutes as described previously[2].

Macrophage cell surface phenotype was assessed by incubating with TruStain FcX Fc receptor blocking solution (422301), True-Stain Monocyte Blocker (426101), CD64-PE-Cy7 (399507), CD206-APC (321109), CD83-PE (305307) (all Biolegend) and fixable near-IR live/dead stain (Thermo Fisher, L34975). Samples were analysed on a Fortessa X20 flow cytometer (BD).

Fast-ATAC-seq and RNA-seq: CD14$^+$ monocytes were enriched from cryopreserved PBMNC samples by MACS for use in both fast-ATAC-seq and RNA-seq. Thirteen CMML patients and three controls were included in these experiments. 5000 cells were used per reaction. Cells were resuspended in 50 µL transposase mixture (1xTD buffer [Illumina, 15027866 or Diagenode, C01019042-1], 2.5 µL TDE1 enzyme [Illumina, 15027865 or Diagenode, C01070012], 0.01% digitonin [Promega, G9441]) after MACS enrichment, and immediately incubated at 37°C for 40 min with 300 rpm agitation. Samples were then purified using a MinElute PCR Purification kit (QIAGEN, 28004) following the manufacturer's instructions. Library preparation was performed as per Buenrostro *et al.*[3] Primers containing adapter sequences can be found in Buenrostro *et al*.[4] The optimal number of PCR cycles was determined for each library separately using a qPCR side reaction as previously described.

Libraries were purified using a MinElute PCR Purification kit (QIAGEN, 28004) according to the manufacturer's instructions. Quality of the libraries was assessed using a Bioanalyzer 2100 and 2100 Expert software (Agilent). Libraries were not size selected prior to sequencing.

RNA was isolated using the RNeasy Micro or Mini kit (QIAGEN), according to manufacturer's instructions. Library preparation was performed using a SureSelect polyA kit (Agilent). All samples were sequenced on a NextSeq 500 (Illumina), generating paired-end reads.

ChIP-seq: $CD14^+$ monocytes isolated from fresh blood were used for this experiment, at $1x10^6$ cells per IP. Five CMML patients and three controls were included. Cells were crosslinked with 1% formaldehyde and the reaction was quenched with glycine. Dynabeads protein G (Thermo Fisher Scientific) were washed with cold IP-100 buffer (25 mM Tris-HCl pH 7.5, 100 mM KCl, 5 mM $MgCl_2$, 10% v/v glycerol, 0.1% NP-40 alternative [Calbiochem, 492016-100ML], 200 µM PMSF) and incubated with antibody for 6-8h at 4°C while rotating. Cells were then sonicated in sonication buffer (50 mM HEPES-KOH pH 7.5, 140 mM NaCl, 1 mM EDTA, 1% v/v Triton X-100, 0.1% w/v sodium deoxycholate, 1x EDTA-free proteinase inhibitor cocktail [PIC] [Roche], 1% v/v SDS) to a fragment size of approximately 500 bp. Sonication buffer without SDS was added to dilute SDS to 0.1% v/v. Antibody-coated beads were added to the rest of the sample, and IP was carried out O/N at 4°C while rotating.

Target	Manufacturer	Catalogue number
H3K4me1	Diagenode	C15410037
H3K4me3	Diagenode	C15410003-10
H3K27ac	Abcam	4729
H3K27me3	Diagenode	C15410195

Beads were washed as follows: Twice with sonication buffer without SDS, twice with wash buffer A (50mM HEPES-KOH pH 7.5, 500 mM NaCl, 1 mM EDTA, 1% v/v Triton X-100, 0.1% w/v sodium deoxycholate, 0.1% v/v SDS, 1x PIC), twice with wash buffer B (20 mM Tris-HCl

pH 8.1, 1 mM EDTA, 250 mM LiCl, 0.5% v/v IGEPAL CA-630, 0.5% w/v sodium deoxycholate, 1x PIC) and twice with TE buffer (10 mM Tris-HCl pH 8.1, 1 mM EDTA), followed by addition of elution buffer (50 mM Tris-HCl pH 8.1, 1 mM EDTA, 1% v/v SDS). Samples were vortexed and incubated at 65°C, after which the first eluate was taken. The elution process was then repeated. NaCl and RNase A were added, and samples were incubated O/N at 65°C. EDTA and proteinase K were added, and samples were incubated at 42°C for 2h. Extraction with 25:24:1 phenol:chloroform:isoamyl alcohol (Sigma, P2069-400ML) was performed twice. Glycogen, sodium acetate pH 5.6 and two volumes of 100% ice-cold ethanol were added, after which DNA was precipitated at -20°C. DNA was washed and then resuspended in 10 mM Tris-HCl pH 7.5. Library preparation was performed using the MicroPlex library preparation kit (Diagenode). Samples were sequenced on a HiSeq 2500 (Illumina) or NextSeq 500, generating paired-end reads. Data from three runs (each comprising all samples) were combined.

Analysis of ATAC-seq and ChIP-seq data: For both ChIP-seq and ATAC-seq data, quality control of fastq files was performed using FastQC[5] (v0.11.7) and multiQC[6] (v1.7). BBDuk (BBTools suite[7], v38.23) was used to trim low-quality reads. Reads were aligned to the hg38 reference genome using bwa-mem[8] (v0.7.17), and duplicates were removed after alignment with Picard tools[9] (v2.19.0). Y-chromosomal and mitochondrial reads were removed from ATAC-seq data using Samtools (v1.9). In addition to quality trimming, adapter trimming was applied to ATAC-seq reads. Adapter trimming was performed with BBDuk, using Trimmomatic's default list of adapters.

ATACseqQC was used to generate plots of insert sizes for ATAC-seq data. Inspection of these plots revealed the presence of artefacts of size 19-21 bp in certain samples, but not others. Inspection of these artefacts in the Integrative Genome Browser (IGV)[10, 11] revealed that they were the result of soft-clipping by bwa and did not in fact align to the genomic positions they had been assigned to. For that reason, they were removed with Samtools (v1.9)[12].

ChIP-seq peaks were called with MACS2[13] (v2.1.2). For H3K27me3, broad peaks were called; for H3K27ac, H3K4me1 and H3K4me3, narrow peaks and summits were called. For ATAC-seq analysis, the flag --no-lambda was set. Intersection of peak sets and removal of blacklist regions (ENCODE annotation file set ENCFF419RSJ) was done using bedtools intersect (v2.27.1-7)[14]. The DAC Blacklisted Regions track was downloaded from the ENCODE project website at https://www.encodeproject.org/files/ENCFF419RSJ/. Peaks were visualised using IGV (v2.4.11). To prepare bigwig files for visualisation, scale factors were calculated as 1/(library size * fraction of reads in peaks). Bigwig files were then created using deepTools (v3.5.1) bamCoverage. Quality control was performed with deepTools[15], using computeMatrix (in reference point mode), plotHeatmap and plotFingerprint.

For differential binding/accessibility analysis, DiffBind[16] (v2.14; R v3.6.2) was used. DiffBind was also used to calculate fractions of reads in peaks. To annotate differentially bound regions as identified by DiffBind to genes, Homer (v 4.10) annotatePeaks.pl was used, again with the hg38 assembly of the human reference genome. Normalisation was done by fraction of reads in peaks.

Super enhancers(SE) were called with Rank Ordering of Super-Enhancers (ROSE, 2013)[17, 18] using the hg38 reference genome, default stitching distance (12.5 kb) and a TSS exclusion zone of 2500. SEs that overlapped with differentially H3K27ac-bound regions were identified using bedtools intersect.

Homer (v4.10) findMotifsGenome.pl was used to identify transcription factor motifs in differentially accessible regions. For this analysis, peak summits extended to width 251 bp were used. These narrow peaks were then used for differential analysis, and the resulting list of differentially accessible/marked regions was passed to Homer with flag -size given set.

Analysis of RNA-seq data: Quality control of fastq files was performed using FastQC[5] (v0.11.7) and multiQC[6] (v1.7). BBDuk (BBTools suite[7], v38.23) was used to trim low-quality reads. Adapters were trimmed with BBDuk’s default list of adapters. Reads were aligned to

the hg38 reference genome with STAR[19] (v2.7.1) with flag --twopassMode Basic set to account for the potential occurrence of novel splice junctions in the CMML samples, given the known prevalence of splicing mutations in CMML[20]. DESeq2[21] was used to identify differentially expressed genes. Gene information was obtained from the Ensembl database, version 86 (hg38, R library EnsDb.Hsapiens.v86)[22], and Y-chromosomal genes were removed from the analysis to correct for the fact that all controls in this dataset were female. DESeq2 analysis was performed as per the manual, using an adjusted p-value cut-off of 0.05. This identified 879 differentially expressed genes (DEGs) between CMML patients and controls. To calculate fragments per kilobase of exon per million mapped fragments (FPKM) values, the function getGeneLengthAndGCContent from the EDAseq[23] R package was used. This function returns the sum of the non-overlapping exonic gene lengths as the gene length. This can then be entered into DESeq2's fpkm() function.

For gene set enrichment analysis (GSEA), GSEA (v.4.3.3) was used[24, 25]. The FPKM values calculated with DESeq2 were used as input. Because the number of samples in the control group was too small for phenotype permutation to be used to calculate significance, gene set permutation was used instead. 1000 permutations were used. For consistency, the same settings were used when analysing publicly available data sets[26, 27]. For pathway analysis using DEGs, the DESeq2 results were used as input to Ingenuity Pathway Analysis (IPA, QIAGEN, v2022.4), using the March 2020 version of the database. IPA was also used for all other pathway analyses.

Mitochondrial stress test: CD14$^+$ monocytes isolated from fresh blood were seeded in a 96-well Seahorse microplate coated with 50 µg/mL PDL (Gibco, A3890401) in a total volume of 80 µL per well, at a density of 125,000 or 250,000 cells per well. At least 4, and ideally 6, technical replicates were used per sample. The plate was allowed to rest for 1h at RT before it was moved to a 37°C tissue culture incubator. Cells were cultured O/N after MACS enrichment to allow them to settle prior to the Seahorse assay. The manufacturer's protocol for the Mito Stress Test Kit (103015-100) was followed, with the addition of a centrifugation step prior to washes to minimise cell loss. The CyQUANT Cell Proliferation Assay (Thermo Fisher, C7026) was used as a proxy to normalise for cell number.

MitoTracker Red-CMXRos (Invitrogen, M7512) and MitoTracker Green-FM (Invitrogen, M7514) were used to stain mitochondria in live cells, in parallel with the mitochondrial stress tests. Cells were stained with CD14-APC (Miltenyi, 130-113-143) and CD16-APC-H7 (BD, 560195) to check monocyte purity after MACS enrichment. Cells were analysed on an LSR II flow cytometer (BD).

qRT-PCR: The high-capacity cDNA reverse transcription kit (Applied Biosystems; 4368814) was used to generate cDNA from RNA. RNase inhibitor (N8080119; Applied Biosystems) or RNasein (Promega, N2511) was included in the cDNA mix. SYBR Green PCR Master Mix (Applied Biosystems, 4309155) was used according to the manufacturer's instructions.

The following primers were used:

CCL22: CGTGATTACGTCCGTTACCGT, CCTGAAGGTTAGCAACACCA;

CD200R: ATCTTCTTAGTGGCCGAAGC, TCCTTGCTAGTTTGCAGCATT;

TGM2: CACTTGGAGGGTCTCGCC, TCTCTAAGACCAGCTCCTCG;

CXCL10: GTCCACGTGTTGAGATCATTGCT, TCGATTTTGCTCCCCTCTGGT;

SOCS1: CTTCCGCACATTCCGTTCGC, CCCCCAGTAGAATCCGCAGG;

IDO1: TTTGCTAAAGGCGCTGTTGGA, GGTTGCCTTTCCAGCCAGACA;

ACTB: AAGTCCCTTGCCATCCTAAAA, ATGCTATCACCTCCCCTGTG;

IL10: TGCCTTCAGCAGAGTGAAGA, GCAACCCAGGTAACCCTTAAA;

IL1B: AGCTCGCCAGTGAAATGATGG, TGGTCGGAGATTCGTAGCTGG;

TNF: CTGCACTTTGGAGTGATCGG, TCAGCTTGAGGGTTTGCTAC;

IL6: GCCCAGCTATGAACTCCTTCT, GCGGCTACATCTTTGGAATC;

IL1A: AACCAGTGCTGCTGAAGGA, TTCTTAGTGCCGTGAGTTTCC;

LIF: GGCCCGGAGAAGAGTATGTA, TCACCACTCCAACAATGACAG;

ACTB: CCAACCGCGAGAAGATGA, CCAGAGGCGTACAGGGATAG;

GAPDH: CCCCGGTTTCTATAAATTGAGC, CTTCCCCATGGTGTCTGAG.

Statistical analysis: Due to small sample sizes (<10 samples per group), non-parametric statistical tests were used. To compare the medians of two populations, such as when comparing fold changes in mean fluorescence intensity in the MitoTracker assays, the Mann-Whitney U test was used. For comparison of the Th2/Th1 ratios, an unpaired t-test with Welch's correction was used due to larger sample numbers but unequal variance across groups (as revealed by an F-test). These tests were performed in GraphPad Prism 9. Correlation analysis was performed in R v3.6.2 using Pearson's correlation coefficient.

Supplementary Figures:

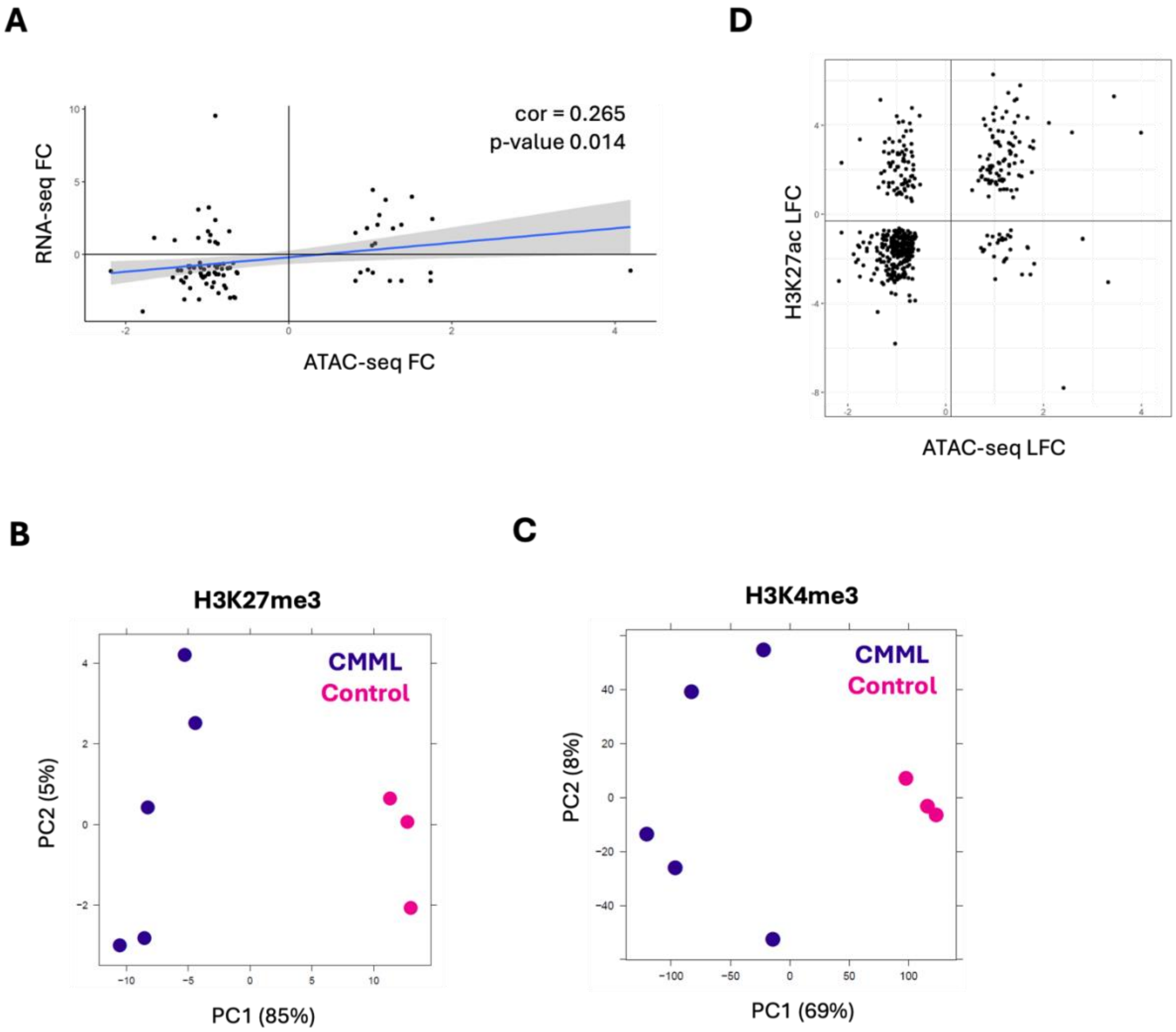

Supplementary Figure 1: A. Correlation between log2 fold change (LFC) in DARs and LFC in DEGs. Pearson's correlation test. **B&C.** PCA plots for H3K27me3 (B) and H3K4me3 (C) ChIP-seq data. **D.** Correlation Plot of LFC in H3K27ac DBRs versus LFC in DARs. Duplicates were resolved by choosing the differentially bound/accessible region with the lowest FDR. DARs: Differentially Accessible Regions; DBR: Differentially Bound Regions.

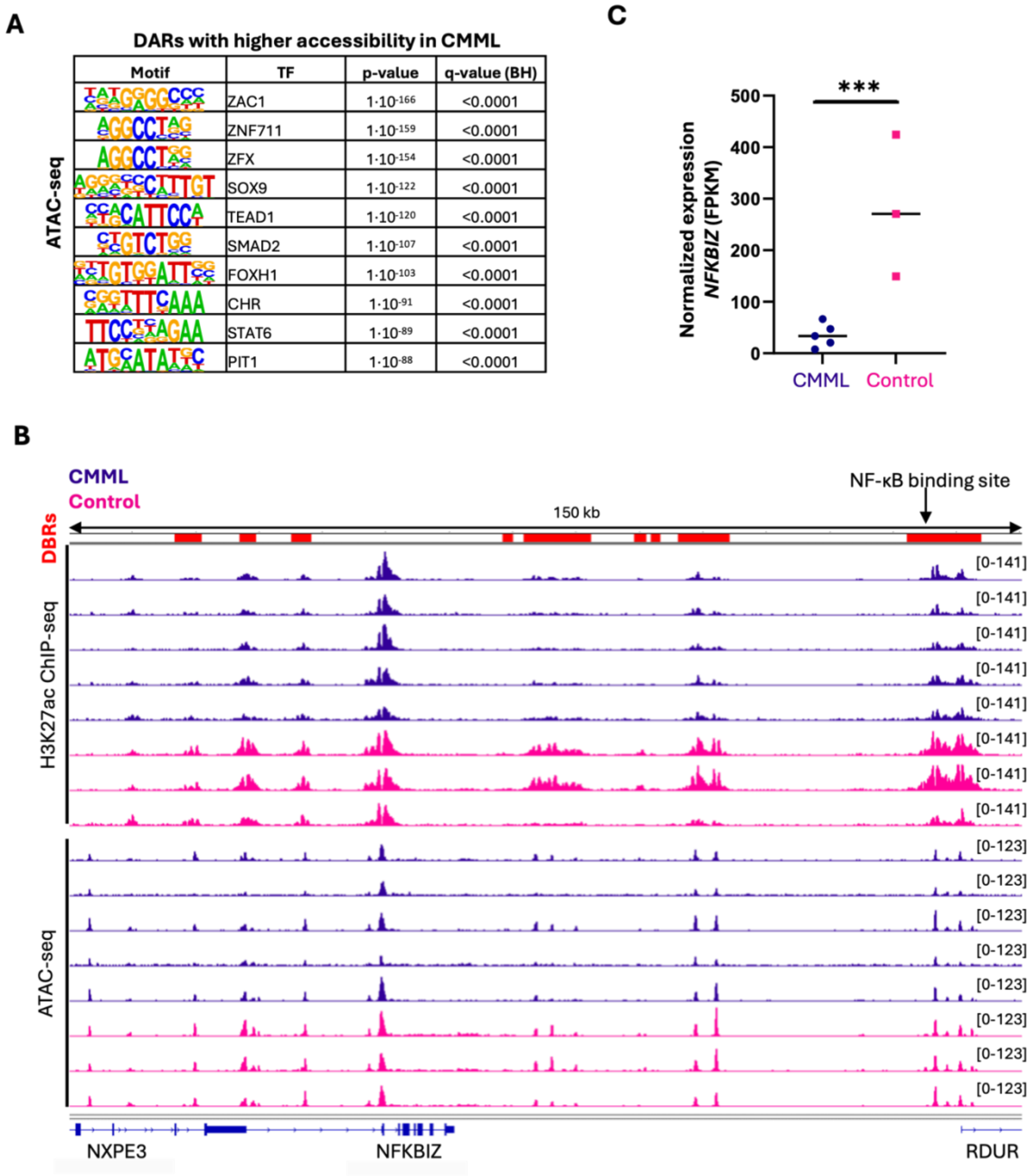

Supplementary Figure 2: A. Motifs most significantly enriched in DARs with higher accessibility in CMML compared to healthy controls. TF = transcription factor; BH = Benjamini-Hochberg correction. **B.** Genome browser plots of the *NFKBIZ* SE, showing H3K27ac ChIP-seq (top) and ATAC-seq for matching samples (bottom). H3K27ac DBRs are marked in red above the tracks, and an NF-κB binding site is indicated. **C.** Expression of *NFKBIZ* as determined by RNA-seq in samples included in the ChIP-seq dataset. SE: Super Enhancer.

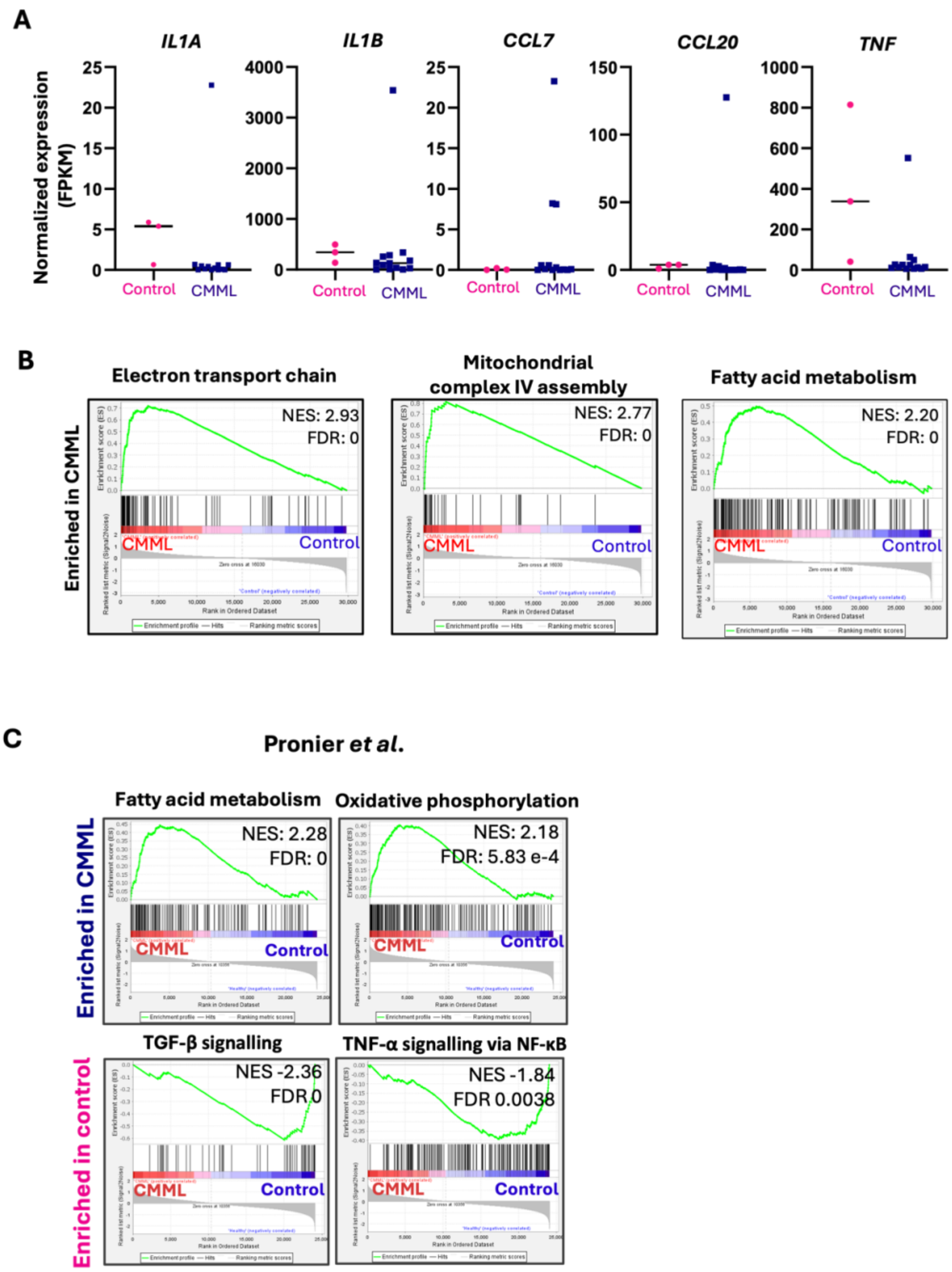

Supplementary Figure 3: A. Expression of selected genes in the cytokine/chemokine signature described by Franzini *et al.*[26] in the current RNA-seq dataset. **B.** Selected pathways identified by GSEA as highly enriched in CMML monocytes. The Wiki pathways and Hallmarks gene set databases were queried. NES = normalized enrichment score. **C**. GSEA analysis of publicly available Pronier *et al.*[27] transcriptomic data. The Hallmarks gene set database was used, and gene set permutation was used for consistency between analyses. Selected pathways found to be enriched in CMML (top) or control samples (bottom) are shown.

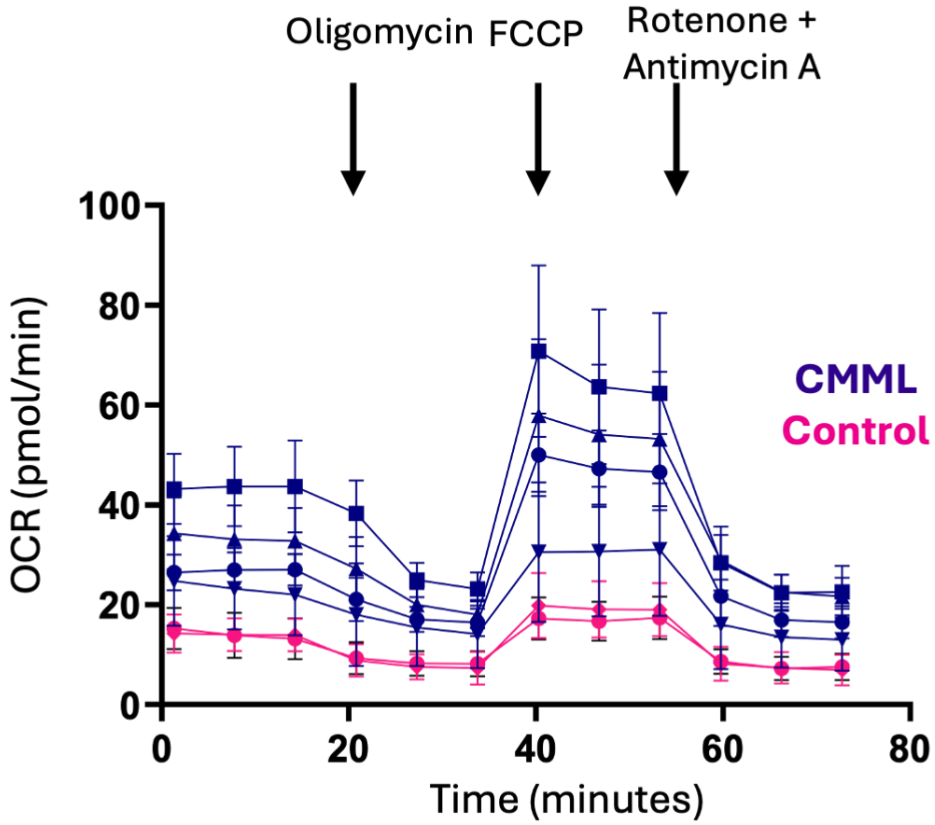

Supplementary Figure 4: Mitochondrial stress test. Data were normalised using DNA content as determined by CyQUANT assay and multiplied by a scale factor.

A

Pathway	-log(p-value)
Macrophage classical activation signalling pathway	8.3
Neuroinflammation signalling pathway	6.8
Pathogen-induced cytokine storm signalling pathway	6.27
Hypercytokinemia/hyperchemokinemia in influenza pathogenesis	5.66
Immunogenic cell death signalling pathway	5.48
Tumor microenvironment pathway	4.85
IL-10 signalling	4.83
Toll-like receptor signalling	4.24
Multiple sclerosis signalling pathway	3.89
Crosstalk between dendritic cells and natural killer cells	3.76
Th1 pathway	3.58

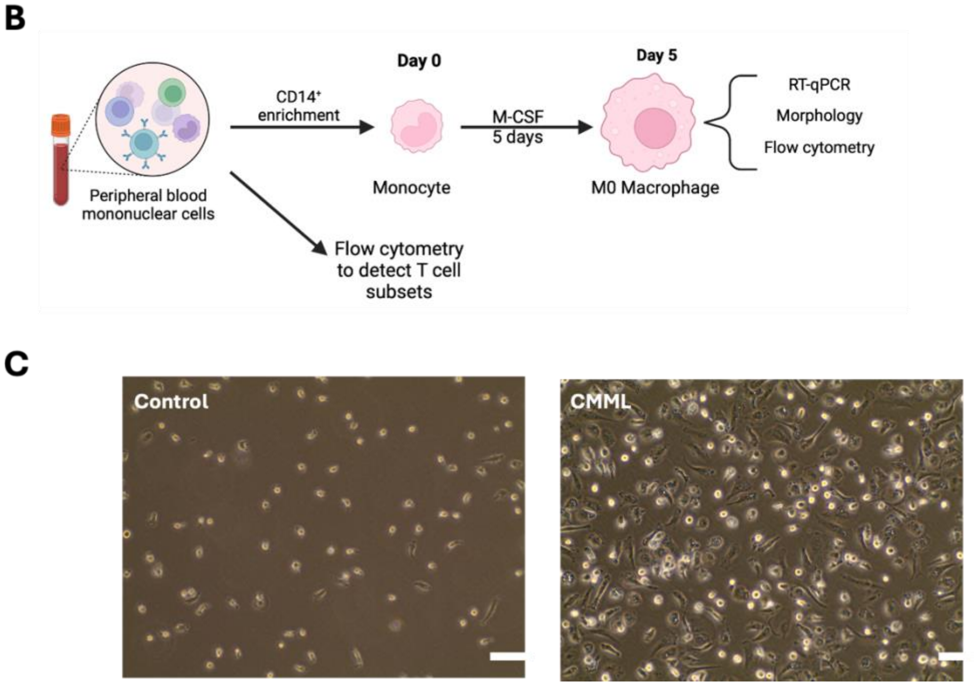

Supplementary Figure 5: A. IPA analysis on genes that map to DARs having NF-κB binding sites and low accessibility in CMML. **B.** Schematic representation of experimental outline to investigate macrophage polarization in CMML monocytes. **C.** Brightfield microscopy images of day 5 MDMs from a representative healthy volunteer (left) and CMML patient (right). Scale bar, 100 µm. The brightness in the right-hand image (CMML) has been increased by 20% to allow for comparison between the two images. MDM: Monocyte Derived Macrophage.

References:

1. Biotec M. Flow analysis of Th subsets guidelines. 2015; 2015. p. 1-3.

2. Chen S, So EC, Strome SE, Zhang X. Impact of Detachment Methods on M2 Macrophage Phenotype and Function. *Journal of Immunological Methods* 2015; **426:** 56-61.

3. Buenrostro JD, Wu B, Chang HY, Greenleaf WJ. ATAC-seq: A method for assaying chromatin accessibility genome-wide. Curr. Protoc. Mol. Biol. Hoboken, NJ, USA: John Wiley & Sons, Inc.; 2015. p. 21.29.21-21.29.29.

4. Buenrostro JD, Giresi PG, Zaba LC, Chang HY, Greenleaf WJ. Transposition of native chromatin for fast and sensitive epigenomic profiling of open chromatin, DNA-binding proteins and nucleosome position. *Nature Methods* 2013; **10:** 1213-1218.

5. Andrews S. *FastQC: a quality control tool for high throughput sequence data*, 2010.

6. Ewels P, Magnusson M, Lundin S, Käller M. MultiQC: Summarize analysis results for multiple tools and samples in a single report. *Bioinformatics* 2016; **32:** 3047-3048.

7. Institute JG. *BBTools*, 2014.

8. Li H. *bwa - Burrows-Wheeler Alignment tool*, 2013.

9. Institute B. *Picard tools*.

10. Thorvaldsdottir H, Robinson JT, Mesirov JP. Integrative Genomics Viewer (IGV): high-performance genomics data visualization and exploration. *Briefings in Bioinformatics* 2013; **14:** 178-192.

11. Robinson JT, Thorvaldsdóttir H, Winckler W, Guttman M, Lander ES, Getz G*, et al.* Integrative genomics viewer. *Nature Biotechnology* 2011; **29:** 24-26.

12. Li H, Handsaker B, Wysoker A, Fennell T, Ruan J, Homer N*, et al.* The Sequence Alignment/Map format and SAMtools. *Bioinformatics* 2009; **25:** 2078-2079.

13. Zhang Y, Liu T, Meyer CA, Eeckhoute J, Johnson DS, Bernstein BE*, et al.* Model-based Analysis of ChIP-Seq (MACS). *Genome Biology* 2008; **9:** R137.

14. Quinlan AR, Hall IM. BEDTools: a flexible suite of utilities for comparing genomic features. *Bioinformatics* 2010; **26:** 841-842.

15. Ramírez F, Ryan DP, Grüning B, Bhardwaj V, Kilpert F, Richter AS*, et al.* deepTools2: a next generation web server for deep-sequencing data analysis. *Nucleic acids research* 2016; **44:** W160-W165.

16. Stark R, Brown G. DiffBind: differential binding analysis of ChIP-Seq peak data. 2011.

17. Whyte WA, Orlando DA, Hnisz D, Abraham BJ, Lin CY, Kagey MH*, et al.* Master transcription factors and mediator establish super-enhancers at key cell identity genes. *Cell* 2013; **153:** 307-319.

18. Lovén J, Hoke HA, Lin CY, Lau A, Orlando DA, Vakoc CR*, et al.* Selective inhibition of tumor oncogenes by disruption of super-enhancers. *Cell* 2013; **153:** 320-334.

19. Dobin A, Davis CA, Schlesinger F, Drenkow J, Zaleski C, Jha S*, et al.* STAR: Ultrafast universal RNA-seq aligner. *Bioinformatics* 2013; **29:** 15-21.

20. Mughal TI, Cross NCP, Padron E, Tiu RV, Savona M, Malcovati L*, et al.* An international MDS/MPN working group's perspective and recommendations on molecular pathogenesis, diagnosis and clinical characterization of myelodysplastic/myeloproliferative neoplasms. *Haematologica* 2015; **100:** 1117-1130.

21. Love MI, Huber W, Anders S. Moderated estimation of fold change and dispersion for RNA-seq data with DESeq2. *Genome Biology* 2014; **15:** 1-21.

22. Rainer J. *EnsDb.Hsapiens.v86: Ensembl based annotation package*, 2017.

23. Risso D, Schwartz K, Sherlock G, Dudoit S. GC-Content Normalization for RNA-Seq Data. *BMC Bioinformatics* 2011; **12:** 480.

24. Mootha VK, Lindgren CM, Eriksson KF, Subramanian A, Sihag S, Lehar J*, et al.* PGC-1α-responsive genes involved in oxidative phosphorylation are coordinately downregulated in human diabetes. *Nature Genetics* 2003; **34:** 267-273.

25. Subramanian A, Tamayo P, Mootha VK, Mukherjee S, Ebert BL, Gillette MA*, et al.* Gene set enrichment analysis: A knowledge-based approach for interpreting genome-wide expression profiles. *Proceedings of the National Academy of Sciences of the United States of America* 2005; **102:** 15545-15550.

26. Franzini A, Pomicter AD, Yan D, Khorashad JS, Tantravahi SK, Than H*, et al.* The transcriptome of CMML monocytes is highly inflammatory and reflects leukemia-specific and age-related alterations. *Blood Advances* 2019; **3:** 2949-2961.

27. Pronier E, Imanci A, Selimoglu-Buet D, Badaoui B, Itzykson R, Roger T*, et al.* Macrophage migration inhibitory factor is overproduced through EGR1 in TET2low resting monocytes. *Communications Biology* 2022; **5:** 110.
